# Editorial: Neuromodulation techniques, mechanisms, and potential benefits for physical activity participation and human performance

**DOI:** 10.3389/fphys.2026.1938460

**Published:** 2026-07-30

**Authors:** Bhushan Thakkar, Daniel Gomes Da Silva Machado, Ehsan Amiri, Edmund O. Acevedo

**Affiliations:** 1 School of Sport, Rehabilitation and Exercise Sciences, University of Essex, Colchester, United Kingdom; 2 Research Group in Neuroscience of Human Movement (NeuroMove), Department of Physical Education, Federal University of Rio Grande do Norte, Natal, Rio Grande do Norte, Brazil; 3 Department of Sport Science, Human Performance Research Center (HPRC), University of Konstanz, Konstanz, Germany; 4 Exercise Metabolism and Performance lab (EMPL), Department of Exercise Physiology, Faculty of Sport Sciences, Razi University, Kermanshah, Iran; 5 Department of Kinesiology and Health Sciences, Virginia Commonwealth University, Richmond, VA, United States

**Keywords:** neural mechanisms, non-invasive brain stimulation, human performance, transcranial electrical stimulation, transcranial magnetic stimulation

## Introduction

Over the last two decades, non-invasive brain stimulation techniques (NIBS), including transcranial magnetic stimulation (TMS) and transcranial electrical stimulation (TES), have gained attention as tools to modulate neural activity and improve our understanding of the central nervous system functioning and its impact on behavior (Huang et al., 2017; To et al., 2018). With regards to its therapeutic or performance enhancement potential in the areas of exercise and sport performance, it has been used in research to enhance motor performance and learning, cognitive training, and neuromuscular control (Hashemirad et al., 2016; Kang et al., 2016; Lefaucheur et al., 2017; Lefaucheur et al., 2020). However, despite the growing body of promising findings, our understanding of the neurobiological mechanisms through which TMS and TES influence brain function in health and disease remains limited.

The purpose of this Research Topic, “Neuromodulation Techniques, Mechanisms, and Potential Benefits for Physical Activity Participation and Human Performance”, was to synthesize and advance the current knowledge on the neurobiological mechanisms impacting the efficacy of neuromodulation in physical activity and athletic performance. This Research Topic brings together 15 articles on NIBS and physical activity, spanning transcranial direct current stimulation (tDCS) in sport-specific and endurance performance, repetitive transcranial magnetic stimulation (rTMS) and peripheral magnetic stimulation in people living with stroke, and tDCS alongside transcutaneous electrical nerve stimulation (TENS), galvanic vestibular stimulation (GVS), and transcutaneous auricular vagus nerve stimulation (taVNS) in exercise and balance. Two review papers complement these studies by summarizing tDCS effects on the neurovascular unit and examining the neural substrates regulating maximal voluntary contraction (MVC), a key marker of maximal strength.

## Efficacy of tDCS on sport-specific endurance performances

The study by Liang et al. assessed the tolerability of active multifocal tDCS (m-tDCS targeting lower-limb specific cortical areas and its efficacy to optimize late-stage performance and phase-specific muscle coordination during an incremental loading cycling exercise in healthy adults compared to sham tDCS. The results of this study indicated that m-tDCS was safe, well tolerated and there was an increase in physiological parameters relative to sham. Additionally, during the propulsion phase, electromyography findings for the quadriceps revealed that muscle contribution ratio of the m-tDCS group was significantly higher than that of the sham. Mechanistically, the authors hypothesized that m-tDCS contributes to changes in neural efficiency, muscle activation, and coordination leading to enhanced endurance. In another study examining neuromuscular coordination, Xiang et al. examined the acute effects of M1- targeted tDCS on golf swing performance. Using a double-blind, randomized, counterbalanced crossover design, they investigated three different tasks requiring long-driving distance capacity and accuracy control in eight professional golfers. The study results demonstrated that active tDCS targeting the M1 region elicited acute and significant improvements in long-driving distance capacity measured using ball speed and carry distance in iron tasks and carry distance in the driver task compared to sham tDCS.

Melahbid et al. were interested in examining the effects of tDCS on women’s recovery and performance. They hypothesized that three sessions of active dual-site tDCS (M1 and L-DLPFC) would enhance both subjective recovery metrics and physical performance in recreationally active females compared to sham stimulation. Performance and recovery were assessed (1) before the tDCS intervention, (2) immediately after a fatigue-inducing time-to-exhaustion test, and (3) following a 24-h recovery period. The study results partially supported the hypothesis with the active tDCS group reporting greater perceived recovery scores on the total quality recovery scale but there were no effects on wellbeing scores, 3-km time trial performance, or explosive power (measured using the Sargent jump test). Additionally, Machado DGS et al., assessed psychophysiological responses (affective valence, arousal, and rating of perceived exertion) and brain oxygenation before and after a 20-min vigorous-intensity exercise session in a cross-over trial where participants received active or sham tDCS targeting the DLPFC region in the brain. The authors concluded that tDCS targeting DLPFC did not influence these outcomes which brings in to focus the role of brain state-dependency when examining the effects of NIBS.

To investigate the feasibility of combining tDCS with aerobic exercise, Abrantes et al. in a pilot randomized trial with 51 participants exhibiting low physical activity levels and elevated depressive symptoms, revealed that the active tDCS group demonstrated an increase in accelerometer-derived daily steps compared to sham. Additionally, both active and sham groups reported decreases in depressive symptoms and increases in cardiorespiratory fitness. This study highlighted the focus of tDCS as an adjunctive treatment to aerobic exercise to enhance exercise outcomes. In a preclinical study in rodents, Van Boekholdt et al., utilized the passive avoidance task (PAT) in combination with tDCS to evaluate memory performance. Improved passive avoidance learning (memory) was observed after transcutaneous-only tDCS of 2mA mimicking the peripheral nerve activation observed in human tDCS experiments comparing transcutaneous-only tDCS, transcranial-only tDCS, and sham. assessed how changes in tDCS dosage and stimulation parameters impacts corticospinal excitability (CSE) to evaluate the mechanisms of changes in exercise performance with the application of tDCS. With regards to tDCS timing before exercise, CSE typically peaks approximately 15–30 min post-stimulation, although concurrent application of tDCS and exercise has variable effects on CSE measurements.

## Effects of repetitive TMS and peripheral magnetic stimulation in people living with stroke

Focusing on central mechanisms of NIBS, Yu et al. used electroencephalography (EEG) and EMG to identify the cortical and neuromuscular changes pre and post rTMS in people living with stroke. Post rTMS, there was an increase in upper limb muscle activity via enhanced motor unit recruitment and synchronization, leading to more forceful and coordinated muscle contractions. Some of the EEG findings correlated with EMG changes suggesting the potential for rTMS to be used as a tool for neurorehabilitation. In a case report addressing hemiplegia in a stroke patient, Itoh et al., studied the effects of repetitive peripheral magnetic stimulation on the extensor muscles of the forearm. The patient received 9 sessions of sham stimulation first followed by active stimulation in combination with routine physiotherapy. Although improvement was observed after treatment, the risk of a placebo response and the inherent limitations with a case report must be considered.

## Research examining effects of different NIBS approaches on exercise and balance performance

In another review in people with chronic ankle instability, Zheng et al. concluded that tDCS combined with exercise did not significantly improve static or dynamic balance compared to exercise alone. In a study measuring changes in postural stability, Vidovic et al. hypothesized that the application of TENS over the lower limb muscles facilitates muscle activation, muscle steadiness and reduces fatigue. They concluded that only TENS applied to the triceps surae muscle reduced postural sway in the mediolateral direction under the eyes closed condition compared to the TENS application over the tibialis anterior muscle. In a narrative review exploring the mechanisms of tDCS on the neurovasculature unit (astrocytes, microglia, and oligodendrocytes), Lewis et al. observed that tDCS induces neuronal growth, plasticity, and improved energetic efficiency. Interestingly, at the level of the synapse, it adjusts protein expression to support sustained plasticity and memory formation. Furthermore, microglial and astrocyte activation cause long-term synaptic effects contributing to neuroprotection and neuroplasticity.

Looking at other forms of TES, Matsuoka et al., concluded that taVNS was a safe technique in healthy individuals and in clinical populations. With regards to its mechanisms, taVNS activates afferent projections from the auricular branch of the vagus nerve to the nucleus tractus solitarius which modulates activity in different brain regions that control cognition, motor control, and emotional regulation.

To examine the effects of GVS, Fu et al., conducted a systematic review and concluded that GVS improves postural control in Parkinsons disease, stroke-induced hemiplegia and bilateral vestibulopathy. The authors also noted considerable variability in GVS protocols, small sample size, and 20% of studies demonstrated high risk of bias. Additionally, they also observed that stochastic resonance, where the addition of noise to a system enhances the system’s response to weak periodic signals, instead of perturbing its effectiveness, was the primary mechanism contributing to effects of GVS. Finally, Takarada created a conceptual framework to probe the neurophysiological contributions to maximal voluntary contraction (MVC), an indicator of maximal strength in isometric muscle assessment. Interventions leading to enhanced activity within the reward-associated dopaminergic system and activation of the pupil-linked neuromodulatory system, could positively modulate MVC. Additionally, they observed that motivational cues like verbal encouragement, actions and words causing unconscious goal pursuit can enhance MVC.

Research questions focused on the role of the central nervous system in health and performance continue to proliferate and move forward with enhanced scientific rigor. The research utilizing NIBS has broaden to include participants from clinical populations, apparently healthy populations, and high-level performers. Importantly, the present Research Topic contributed to meaningful advancement and enhanced understanding of the neural mechanisms linked to the efficacy of TMS, TES and other NIBS techniques in the areas of physical activity and athletic performance. However, it is clear that much is left to be investigated and clarified to better under which type of NIBS affect or do not affect a specific clinical population or group of individuals. Hypothesis driven studies examining possible mechanisms linked to central and peripheral nervous system function associated with patient populations and human performance will lead to a greater understanding of the benefits of NIBS. More specifically, studies should consider focusing on dual-site and/or network-based approaches, targeting regions such M1, DLPFC, the posterior parietal cortex and/or cerebellum, to address the full complexity of physical activity behavior. Finally, it is critical to develop studies that incorporate the role of the brain state to reduce NIBS variability for biomarker discovery and therapeutic interventions.
