# Correction: Editorial: Neuromodulation techniques, mechanisms, and potential benefits for physical activity participation and human performance

**DOI:** 10.3389/fphys.2026.1964921

**Published:** 2026-08-26

**Authors:** Bhushan Thakkar, Daniel Gomes Da Silva Machado, Ehsan Amiri, Edmund O. Acevedo

**Affiliations:** 1 School of Sport, Rehabilitation and Exercise Sciences, University of Essex, Colchester, United Kingdom; 2 Research Group in Neuroscience of Human Movement (NeuroMove), Department of Physical Education, Federal University of Rio Grande do Norte, Natal, Rio Grande do Norte, Brazil; 3 Department of Sport Science, Human Performance Research Center (HPRC), University of Konstanz, Konstanz, Germany; 4 Exercise Metabolism and Performance lab (EMPL), Department of Exercise Physiology, Faculty of Sport Sciences, Razi University, Kermanshah, Iran; 5 Department of Kinesiology and Health Sciences, Virginia Commonwealth University, Richmond, VA, United States

**Keywords:** neural mechanisms, non-invasive brain stimulation, human performance, transcranial electrical stimulation, transcranial magnetic stimulation

Affiliation “Exercise Metabolism and Performance lab (EMPL), Department of Exercise Physiology, Faculty of Sport Sciences, Razi University, Kermanshah, Iran” was omitted from the paper. This affiliation has now been added for author(s) “Ehsan Amiri”.

The original version of this article has been updated.

